# Randomized Controlled Study of Sirolimus-Coated Balloons for Dysfunctional Hemodialysis Fistulas

**DOI:** 10.1016/j.ekir.2026.106583

**Published:** 2026-05-08

**Authors:** Chieh Suai Tan, Ru Yu Tan, Pei Ho, Edward Tieng Chek Choke, Chee Wooi Tan, Alvin Ren Kwang Tng, Gek Hsiang Lim, Hsien Ts’ung Tay, Kun Da Zhuang, Jun Jie Ng, Kalpana Vijaykumar, Darius Kang Lie Aw, Tze Tec Chong, Kiang Hiong Tay, Suh Chien Pang, IMPRESSION investigators

**Affiliations:** 1 Department of Renal Medicine, Singapore General Hospital, Singapore; 2 Department of Surgery, Yong Loo Lin School of Medicine, National University of Singapore, Singapore; 3 Department of Cardiac, Thoracic and Vascular Surgery, National University Health System, Singapore; 4 Department of Surgery, Sengkang General Hospital, Singapore; 5 Health Services Research Unit, Singapore General Hospital, Singapore; 6 Department of Vascular Surgery, Singapore General Hospital, Singapore; 7 Department of Vascular and Interventional Radiology, Singapore General Hospital, Singapore

**Keywords:** angioplasty, dialysis access, sirolimus-coated balloon

## Abstract

**Introduction:**

Restenosis following balloon angioplasty remains a major cause of arteriovenous fistula (AVF) dysfunction in patients receiving maintenance hemodialysis. We evaluated whether the use of sirolimus-coated angioplasty balloons (SCBs) improves access circuit primary patency (ACPP) in patients with dysfunctional, nonthrombosed AVFs.

**Methods:**

In this investigator-initiated, multicenter, single-blinded, randomized controlled trial (ClinicalTrials.gov identifier: NCT04409912), we enrolled 170 patients from 3 tertiary teaching hospitals in Singapore. Patients with dysfunctional AVFs, including those with single or multiple stenotic lesions within the access circuit, were included. Following successful plain balloon angioplasty (PBA) (< 30% residual stenosis on digital subtraction angiography), the patients were randomly assigned, using a centralized web-based system, to receive additional treatment with SCB (*n* = 83) or placebo balloons (*n* = 87). During the 12-month follow-up, the patients and their managing physicians were blinded to the study treatment and placebo. The proceduralists and coordinators could not be blinded because of visible differences between the balloons. The primary end point was ACPP during the first 6 months after the index procedure. Secondary end points included 12-month patency and safety outcomes.

**Results:**

In both intention-to-treat (ITT) and per-protocol (PP) analyses, 6-month ACPP was significantly higher in the SCB group than in the placebo group (*P* = 0.03). At 6 months, 70.1% (95% confidence interval [CI]: 58.7–78.8) of patients treated with SCB remained patent, compared with 56.7% (95% CI: 45.5–66.4) in the placebo group. At 12 months, the difference in patency was not statistically significant in the ITT analysis (37.1% vs. 27.7%; *P* = 0.07) but remained significant in the PP analysis (36.4% vs. 25.2%; *P* = 0.04). Adverse events were infrequent and no deaths occurred within 30 days in both groups.

**Conclusion:**

Use of SCB following successful PBA improved 6-month ACPP in patients with dysfunctional AVFs, including those with multiple stenotic lesions. Short-term safety findings were reassuring.

## Graphical abstract

**Figure:**
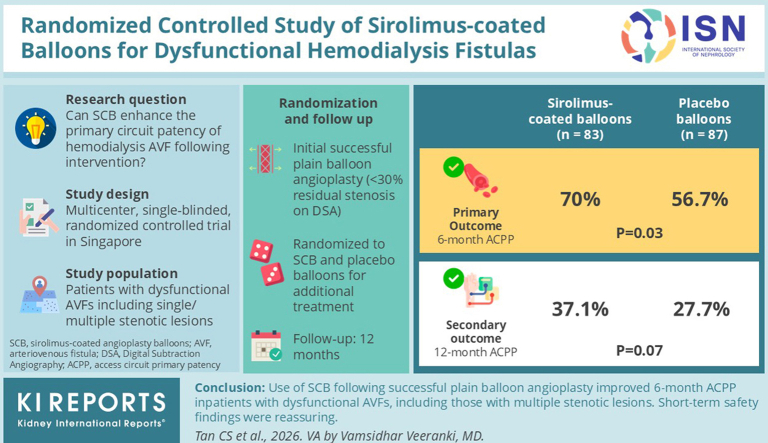

Arteriovenous fistula (AVF) is the preferred choice of vascular access for patients with end-stage kidney failure undergoing hemodialysis because it has a lower risk of infection and superior patency rate than arteriovenous grafts and catheters.^1^ However, stenosis secondary to neointimal hyperplasia often develops within the AVF, resulting in vascular access dysfunction, inadequate dialysis, and thrombosis.^2^^,^^3^ Percutaneous transluminal angioplasty (PTA) with plain balloons is currently the recommended intervention for managing dysfunctional AVFs because of its minimally invasive nature.^1^^,^^4^

Despite the high technical success rate of PTA in treating dysfunctional AVFs, the 6- month access circuit primary patency (ACPP) rates after intervention are approximately 43.8% to 57%^5^, ^6^, ^7^ in the PTA arm of recent large randomized controlled studies. The limited long-term patency with PTA was attributed to endothelial denudation caused by the mechanical dilatation of stenosis with angioplasty balloons.^8^ Consequently, neointimal hyperplasia as a result of PTA resulted in a vicious cycle of stenosis, angioplasty-induced vascular injury, and restenosis.^9^^,^^10^ Therefore, multiple PTAs are often necessary to sustain AVF patency throughout the lifetime of a hemodialysis patient.^11^

In recent years, paclitaxel-coated balloons (PCBs) have been used to improve the patency of the AVFs after PTA. The transfer of paclitaxel, an antiproliferative agent used in the treatment of malignancy, from the surface of a PCB to the vessel wall during balloon inflation is hypothesized to inhibit angioplasty-induced neointimal hyperplasia and therefore improve the patency of the AVF after PTA.^12^^,^^13^ Results have however been mixed, with only 8 out of 21 randomized controlled studies demonstrating the superiority of PCBs over plain balloon PTA in a narrative analysis.^14^ Variability in end point selection, balloon deployment techniques, paclitaxel dosages, and excipient types have been postulated as potential reasons for these discrepancies.^15^, ^16^, ^17^, ^18^ Furthermore, many of the multicenter randomized controlled studies recruited only patients with a single target lesion or tandem lesions that are < 10 cm in length that can be treated with a single PCB.^5^^,^^19^, ^20^, ^21^, ^22^ In clinical practice, multiple stenoses within the dialysis circuit can be present^23^^,^^24^ and the optimal treatment in such patients is unclear.

SCBs are new drug-coated balloons designed to deliver sirolimus to the vessel wall during PTA.^25^^,^^26^ In contrast to paclitaxel, which is cytotoxic, sirolimus is an antiproliferative agent with cytostatic properties^27^ and may represent an alternative drug-coated balloon strategy. Clinically, sirolimus is administered orally as an immunosuppressant to prevent organ rejection and is used in the management of immune-related tumors such as Kaposi sarcoma and EBV-related smooth muscle tumors.^28^^,^^29^ SCBs have been used in coronary and peripheral arterial interventions^30^^,^^31^ and have recently shown promising results when used in the management of dysfunctional AVFs and arteriovenous grafts in a few small single-center studies with 6-month ACPP of between 62.9% into 68.0%.^32^, ^33^, ^34^, ^35^, ^36^

We hypothesized that the application of SCBs to all significant stenotic lesions (defined as >50% stenosis), whether single or multiple, in AVFs adequately pretreated with PTA would result in improved 6-month ACPP compared with PTA alone. Given that PTA alone remained the standard of care and PCB had not been established as an unequivocal comparator at the time this study was conceived, the trial was designed as a randomized evaluation of SCB against PTA rather than a larger 3-arm study including a PCB arm. ACPP was used as our primary end point because in clinical practice, all significant stenoses identified during the procedure would be treated with drug-coated balloons with the aim of prolonging the time to next intervention.^37^

## Methods

### Trial Design

This was an investigator-initiated, multicenter, prospective, single-blinded randomized controlled trial conducted in Singapore. Patients with dysfunctional AVF, including those with single or multiple stenotic lesions within the access circuit, were randomized at a 1:1 ratio to receive either SCB or placebo plain balloons following successful PTA of dysfunctional AVF. The rationale and trial protocol had previously been published, and the trial was registered with ClinicalTrials.gov (NCT04409912).^38^ The trial was conducted in compliance with the principles of the Declaration of Helsinki, and the protocol was approved by the Singhealth Institution Review Board (Reference number 2019/2896). All the patients provided written informed consent before undergoing any trial-specific procedures. The data are owned by the individual institutions that participated in the study but shared for the purpose of analysis.

### Trial End Points

The primary end point was ACPP, defined as freedom from clinically driven reintervention or thrombosis of the AVF during the 6 months after the index procedure. The secondary end points included ACPP during the 12 months, AVF flow rates at 3, 6, and 12 months after the index procedure and target lesion patency. Because ACPP depends on clinically driven repeat intervention, standardized prespecified clinical criteria were used. These included thrombosis or partial thrombosis of the AVF; abnormalities in pulse, thrill, or bruit on physical examination; clinical features of inflow or outflow stenosis; new cannulation difficulty; inability to achieve target dialysis blood flow; prolonged bleeding; and unexplained reduction in delivered Kt/V. These criteria were consistent with the Kidney Disease Outcomes Quality Initiative clinical practice guideline for vascular access, 2019 update.^1^ Patients who required repeat interventions of the AVF for these clinically driven indications were assessed to have lost their circuit patency unless there were no stenotic lesions on the diagnostic fistulogram performed during the repeat intervention. Indications for reintervention were systematically reviewed by the Clinical Evaluation Committee.

### Trial Devices

The study balloons (sirolimus-coated [MagicTouch AVF, Concept Medical, India] and placebo balloon) used in both arms were provided by Concept Medical and had the same profile, inflation pressure, and identical packaging. In addition, the balloons were labeled as “A” and “B” to maintain the blinding. All the balloons were of the 0.035" platform and available in diameters of 5, 6, 7, 8, and 12 mm; lengths of 60, 80, and 100 mm; and shaft lengths of 45 or 90 cm. The sirolimus dose on the SCB was 1.27 μg/mm^2^. Drug transfer was facilitated without an excipient through the proprietary Nanolute drug-delivery system by Concept Medical. Submicron phospholipid nanoparticles were used to encapsulate and stabilize sirolimus for controlled release.^39^ If required, more than one study balloon may be used for each patient to ensure adequate coverage of long or multiple lesions.

### Trial Population

The participants were patients aged between 21 and 85 years with mature AVF, defined as being in use for at least 1 month before enrolment, who were referred to our centers for dysfunctional access. These dysfunctional accesses were detected either through the monitoring or surveillance programs in our community dialysis centers. The AVF circuit was defined as the arteriovenous anastomosis (extending ≤2 cm into the inflow artery) through to the central veins. The degree of lesion stenosis in the AVF must be ≥ 50% as determined by angiography and exhibit one of the symptoms such as significantly increased venous or negative access pressures during dialysis, abnormal physical examination, low flow, or inadequate dialysis clearance. The other key inclusion criterion was successful PTA of the underlying stenosis within the AVF, defined as less than 30% residual stenosis on digital subtraction angiography before randomization. Patients with residual stenosis of more than 30% were considered screen failures and excluded from the study. Other key exclusion criteria included patients with thrombosed or partially thrombosed AVF at the time of study entry, history of stent placement within the AVF circuit, and patients with central vein stenosis. Details of the inclusion and exclusion criteria were as published previously.^38^

### Trial Procedure

The participants and their renal physicians and vascular surgeons were unaware of the treatment assignments. Because of the visible differences between the SCB and placebo balloon, the proceduralists and trial coordinators might be aware of the treatment assignment but they were trained to conceal the assignment from the participants.

After enrolment, the patient underwent fistulograms of the entire dialysis circuit from the feeding artery, arteriovenous anastomosis, to the central veins. All stenoses in the AVF were treated in the standard fashion with PBA. When there was more than one stenosis, all the lesions were labeled and treated accordingly. Stenoses were considered separate if they were separated by a gap of ≥ 2 cm. Angioplasty of each lesion was performed with a plain balloon that was sized based on the adjacent reference vessel. Inflation time was ≥1 minute per inflation. If there was significant residual stenosis after the initial angioplasty, repeat angioplasty with a larger diameter, higher pressure, or cutting balloon may be used at the operator’s discretion to achieve a residual stenosis of less than 30%. In the stenotic segment adjacent to an aneurysmal segment, where the percentage of stenosis was difficult to determine, vessel diameter had to reach at least 6 mm to be considered for inclusion.

Once all the target lesions were adequately treated, as visually assessed by the operator to have <30% residual stenosis, the patients were randomized, using a secure independent web-based randomization program developed by the Singapore Clinical Research Institute, to receive repeat angioplasty with SCB or placebo balloons. Randomization was based on the permuted block randomization with the 3 participating sites and location of the AVF (above or below the elbow) as stratification factors. Repeat angioplasty with the study balloons (SCB or placebo) over all the treated stenoses was performed with a balloon inflation time of at least 2 minutes. Specifically, more than one SCB or placebo balloons may be used in AVF with multiple stenoses to ensure that all treated lesions are covered. The 2 minutes duration was arbitrarily chosen because this was the time duration used for PCB trials. To ensure adequate sizing and apposition to the vessel wall for drug delivery, the study balloon diameter was selected according to the diameter of the balloon used for PBA. The balloon length was selected to exceed the target lesion by approximately 10 mm at either end to ensure full coverage and prevent geographic miss. If more than one balloon was needed to cover a long segment stenosis, an overlap of approximately 10 mm was required.

If flow-limiting dissection, rupture, or residual stenosis > 30% was encountered after repeat angioplasty with the study balloons, additional balloon inflation with plain balloon was performed and the patients were analyzed as per ITT in their original allocation.

### Follow-Up

All the participants were followed-up for at least 12 months after the index procedure. The patients were scheduled for follow-up with duplex ultrasonography at 3, 6, and 12 months after the index procedure. Unscheduled visits, including repeat interventions, were captured throughout the study and treated in accordance with the institution’s standard of care. Patients who required repeat intervention on the AVF were considered to have reached the primary end point and no further duplex ultrasonography was performed for subsequent follow-up. The original trial design specified the establishment of a core laboratory; however, this was not implemented because the 3 participating sites used different fluoroscopy systems (Siemens in Singapore General Hospital and National University Hospital, General Electric in Sengkang Hospital), and the vascular analysis modules of the systems were not cross-compatible. To address this limitation, the principal and site principal investigators convened a clinical evaluation committee, which systematically reviewed all images obtained during the trial to ensure compliance with the inclusion and exclusion criteria, verify the accuracy and consistency of data collection, and confirm the appropriateness of indications for reinterventions throughout the study. This approach was adopted to maintain trial integrity and standardization in the absence of a core laboratory.

### Sample Size Calculation

When the study was designed in 2019, the pilot data supported the assumption that the SCB would be as effective as the PCB. In the Lutonix investigational device exemption study, the difference in ACPP between PCB and PBA mirrored the difference in target lesion primary patency,^19^ justifying the use of target lesion data to estimate circuit-level outcomes. A meta-analysis published during the conception of this study reported a 6-month primary patency rate of 73.7% with PCB versus 55.2% with PBA, an absolute difference of 18.5%.^15^ Assuming SCB performs similarly to PCB, we calculated that 170 participants (85 per arm) would be required to detect this difference at 6 months with 80% power and a 2-sided type I error of 5%, allowing for 10% attrition.

### Statistical Analysis

Descriptive statistics were used to summarize the baseline characteristics of patients who received the SCB and placebo balloons respectively. Frequencies (%) were reported for categorical variables, and median (interquartile ranges [IQRs]) were reported for continuous variables because their distributions were skewed.

The ITT approach formed the main analyses and the PP approach formed the secondary analyses whereby patients who withdrew or died before the 12-month primary end point were excluded from the analyses. For the primary effectiveness analyses, the time-to-event method was used to study the effect of SCBs on the loss of ACPP as compared with placebo balloons. The patients were followed-up from the date of index intervention to the date of repeat interventions because of clinically driven indications, or to the date of review with duplex ultrasonography, whichever came earlier. Patients were considered to have an event if they presented with clinically driven indications or were censored otherwise. Kaplan-Meier curves were presented to show the differences in loss of ACPP in patients with SCB or placebo over time. Correspondingly, log rank tests were used for the difference in loss of ACPP between the 2 balloon types. Log rank tests were performed to capture differences in the experiences in ACPP between patients treated with SCB and placebo balloons for the first 6 months (primary end point) and then for the entire 12 months (secondary end point) since randomization. There was no adjustment in the *P*-values for the multiple comparisons, because the primary objective of this study was to examine if there was a difference in ACPP between SCB and placebo balloons over the first 6 months. The study assessed if there was a difference in ACPP between SCB and placebo balloons over 12 months as a secondary objective. For the primary safety analyses, the number of patients with ≥1 adverse event (bleeding/hematoma or venous rupture) within 30 days formed the numerator and the total number of surviving patients at 30 days formed the denominator; and the corresponding proportions were tabulated for SCB and placebo. Secondary safety analyses included tabulating the proportion of patients who had undergone thrombolysis for thrombosed AVF or died within 30 days. The difference in the proportions of patients with adverse events was tested for noninferiority based on the Farrington-Manning test with a margin of 7.5%. All tests were declared statistically significant if a 2-sided *P*-value < 0.05 was reached, unless otherwise stated. All statistical analyses were conducted using Stata 17 (StataCorp 2021. Stata Statistical Software: Release 17. College Station, TX).

## Results

**Figure 1:**
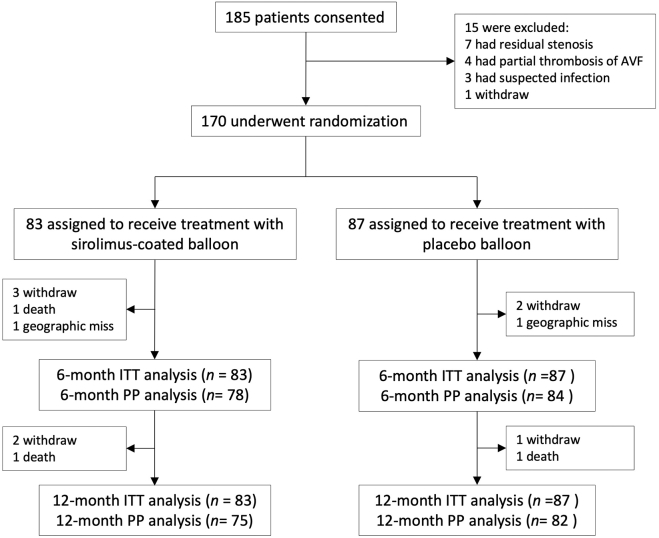
Enrolment, randomization, and follow-up. AVF, arteriovenous fistula; ITT, intention-to-treat; PP, per-protocol.

A total of 170 patients at 3 sites in Singapore underwent randomization in the study; 83 were assigned to receive treatment with SCB and 87 were assigned to receive treatment with a placebo balloon (Figure 1). The baseline characteristics of the study population are shown in Table 1. The demographic characteristics were similar in both groups with an expected high prevalence of diabetes, hypertension, and cardiovascular disease. Antiplatelet and anticoagulant usage was similar in both groups. The distribution between the upper arm (brachiocephalic and brachiobasilic) and lower arm (radiocephalic, ulnar basilic, and snuffbox) AVFs was even. The most common presenting clinical indications for dysfunctional AVF were dropping access flow with accompanying features of abnormal physical examination findings, low flow, difficulties with cannulation, and high venous pressures during dialysis. The majority of the AVFs had multiple stenoses with the juxta-anastomosis being the most common site of stenosis (77.7%), followed by cannulation zone (35.9%), and arteriovenous anastomosis (21.8%). The number of interventions in the 12 months before enrolment was similar in both groups.

**Table 1:** Baseline patient characteristics by treatment group

	Patients with sirolimus-coated balloons (*n* = 83)	Patients with placebo balloons (*n* = 87)	Total number of patients (*N* = 170)	*P*-value
Age of patient at procedure (yrs); median (IQR)	65 (56–71)	65 (59–71)	65 (58–71)	0.66
Duration since ESKD diagnosis (yrs); median (IQR)	2.6 (1.4–5.5)	3.0 (1.5–6.7)	2.8 (1.5–6.1)	0.27
Gender; *n* (%)				0.26
Male	61 (73.5)	57 (65.5)	118 (69.4)	
Female	22 (26.5)	30 (34.5)	52 (30.6)	
Race; *n* (%)				0.80
Chinese	57 (68.7)	59 (67.8)	116 (68.2)	
Malay	20 (24.1)	20 (23.0)	40 (23.5)	
Indian	6 (7.2)	7 (8.1)	13 (7.7)	
Others	0 (0.0)	1 (1.2)	1 (0.6)	
Site of Study, *n* (%)				0.96
Singapore General Hospital	56 (67.5)	57 (65.5)	113 (66.5)	
Sengkang General Hospital	13 (15.7)	14 (16.1)	27 (15.9)	
National University Hospital	14 (16.9)	16 (18.4)	30 (17.7)	
Etiology of ESKD; *n* (%)				0.53
Diabetes mellitus	54 (66.7)	62 (72.9)	116 (69.9)	
Chronic glomerulonephritis	10 (12.4)	9 (10.6)	19 (11.5)	
Polycystic kidney disease	3 (3.7)	2 (2.4)	5 (3.0)	
Hypertension	4 (4.9)	7 (8.2)	11 (6.6)	
Others	10 (12.4)	5 (5.9)	15 (9.0)	
Antiplatelet or antithrombotic therapy; *n* (%)				0.93
None	24 (28.9)	24 (27.6)	48 (28.2)	
Antiplatelet only	50 (60.2)	53 (60.9)	103 (60.6)	
Anticoagulant only	5 (6.0)	7 (8.1)	12 (7.1)	
Both	4 (4.8)	3 (3.5)	7 (4.1)	
Age of AVF (yrs); median (IQR)	2.0 (1.1–5.4)	2.4 (1.3–5.6)	2.1 (1.2–5.4)	0.54
Side of AVF; *n* (%)				0.65
Right	14 (16.9)	17 (19.5)	31 (18.2)	
Left	69 (83.1)	70 (80.5)	139 (81.8)	
Type of AVF; *n* (%)				0.54
Radiocephalic	45 (54.2)	46 (52.9)	91 (53.5)	
Brachiocephalic	22 (26.5)	28 (32.2)	50 (29.4)	
Brachiobasilic	14 (16.9)	13 (14.9)	27 (15.9)	
Others	2 (2.4)	0 (0.0)	2 (1.2)	
Number of target lesions; *n* (%)				0.57
1	36 (43.4)	34 (39.1)	70 (41.2)	
≥ 2	47 (56.6)	53 (60.9)	100 (58.8)	
Number of previous interventions in past 12 mos; *n* (%)				0.85
0	31 (37.4)	31 (35.6)	62 (36.5)	
1	29 (34.9)	34 (39.1)	63 (37.1)	
≥ 2	23 (27.7)	22 (25.3)	45 (26.5)	
Type of last intervention				0.67
Nil	22 (26.5)	18 (20.7)	40 (23.5)	
Angioplasty	59 (71.1)	67 (77.0)	126 (74.1)	
Thrombolysis	2 (2.4)	2 (2.3)	4 (2.4)	
Site of stenosis; *n* (%)				
Feeding artery	1 (1.2)	2 (2.3)	3 (1.8)	0.99
AV anastomosis	15 (18.1)	22 (25.3)	37 (21.8)	0.25
Juxta-anastomosis	62 (74.7)	70 (80.5)	132 (77.7)	0.37
Cannulation zone	34 (41.0)	27 (31.0)	61 (35.9)	0.18
Outflow vein	14 (16.9)	18 (20.7)	32 (18.8)	0.52
Cephalic arch	11 (33.3)	14 (34.2)	25 (33.8)	0.94
Swing zone	6 (33.3)	8 (40.0)	14 (36.8)	0.67
Types of balloons used for vessel preparation; *n* (%)				0.70
Cutting balloons	18 (21.7)	21 (24.1)	39 (22.9)	
High pressure balloons only	65 (78.3)	66 (75.9)	131 (77.1)	
Balloons used for vessel preparation				
Number of balloons/stenosis, median (IQR)	1.5 (1–2)	1 (1–2)	1 (1–2)	0.22
Diameter of balloon (mm); median (IQR)	6 (5.5–6.5)	6 (6–6.5)	6 (5.5–6.5)	0.79
Length of balloon (cm); median (IQR)	6 (4–10)	6 (4–10)	6 (4–10)	0.37
Inflation pressure (atm); median (IQR)	22 (16–24)	21 (16–24)	21.5 (16–24)	0.83
Number of inflations/stenosis; median (IQR)	2 (1–3)	2 (1–3)	2 (1–3)	0.42
Treatment balloons				
Diameter of balloon (mm); median (IQR)	6 (6–7)	6 (6–7)	6 (6–7)	0.80
Length of balloon (cm); median (IQR)	8 (6–10)	8 (6–10)	8 (6–10)	0.83
Inflation pressure (atm); median (IQR)	12 (10–13)	12 (12–14)	12 (11.5–14)	0.16
Number of treatment balloons used per patient; *n* (%)				0.10
1	42 (50.6)	42 (48.3)	84 (49.4)	
2	36 (43.4)	31 (35.6)	67 (39.4)	
≥ 3	5 (6.0)	14 (18.4)	19 (11.2)	

AV, arteriovenous; AVF, arteriovenous fistula; ESKD, end-stage kidney disease; IQR, interquartile range.

Procedural characteristics were as follows: the median number of balloons used for vessel preparation was 1 (IQR: 1–2). The median number of inflations per stenosis for vessel preparation was 2 (IQR: 1–3) with a rated burst pressure of 21.5 (IQR: 16–24) atm. The median diameters and lengths of balloons used for vessel preparation were 6 (IQR: 5.5–6.5) mm × 6 (IQR: 4–10) cm, respectively. Cutting balloons were used in 22.9% of the cases to achieve residual stenosis < 30% before randomization. The median diameters and lengths of the placebo balloons and SCB that were used were both 6 (IQR: 6–7) mm × 8 (IQR: 6–10) cm, with an inflation pressure of 12 (IQR: 10–14) atm. Approximately half of the patients (51.6%) in the SCB and placebo groups had multiple lesions that required >1 trial balloon to cover the treated segment. There were no statistical differences in the types and sizes of balloons for vessel preparation and the sizes of the SCB and placebo balloons in both arms.

### Effectiveness Analysis

**Figure 2:**
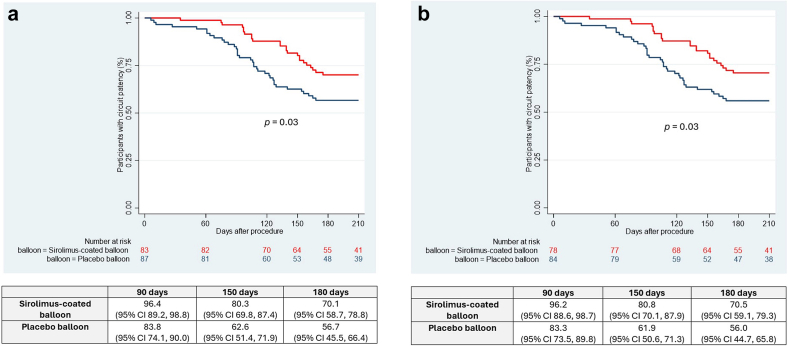
(a) Kaplan-Meier analyses of access circuit primary patency during 6 months after index intervention (ITT analyses). (b) Kaplan-Meier analyses of access circuit primary patency during 6 months after index intervention (PP analyses). CI, confidence interval; ITT, intention-to-treat; PP, per-protocol.

Using the ITT approach, there was a significant difference in the ACPP between SCB and placebo balloons over the first 6 months (*P* = 0.03) as shown in Table 2. At 6 months, 70.1% (95% CI: 58.7–78.8) of the patients treated with SCB remained patent whereas 56.7% (95% CI: 45.5–66.4) of the patients treated with placebo balloons were patent. The differences in ACPP at 6 months were similar using the PP approach (70.5% in SCB vs. 56.0% in placebo). The indications and types of repeat interventions resulting in loss of ACPP are summarized in Supplementary Table S1. In Figure 2a and b, we show the time course of the primary outcomes with cumulative ACPP of the SCB group being significantly higher than that of the placebo group. Over the 12-month follow-up, the SCB group required fewer repeat interventions than the placebo group as shown in Table 2. In addition, 38 patients (45.8%) in the SCB group and 29 patients (33.3%) in the placebo group required no repeat intervention over 12 months, whereas 39.8% and 56.3%, respectively, required 1 to 2 repeat interventions during this period. These findings were consistent with the higher 12-month ACPP observed in the SCB group. The number of thrombolysis performed was lower in patients who received SCB than in those who received the placebo balloon. Despite lower reintervention rates in patients who received SCB over placebo balloons, blood flow rates in the AVF, measured using brachial artery flow rate and in outflow veins at 3, 6 and 12 months postinterventions were similar between both the SCB and placebo groups.

**Table 2:** Primary and key secondary end points

End point	Sirolimus-coated balloon (*n* = 83)	Placebo balloon (*n* = 87)	*P*-value
Primary effectiveness end point			
Access circuit primary patency (ITT analysis) over 6 mos, % (95% CI)	70.1 (58.7–78.8)	56.7 (45.5–66.4)	0.03
Access circuit primary patency (PP analysis) over 6 mos, % (95% CI)	70.5 (59.1–79.3)	56.0 (44.7–65.8)	0.03
Secondary effectiveness end point			
Access circuit primary patency (ITT) over 12 mos, % (95% CI)	37.1 (25.7–48.4)	27.7 (18.2–38)	0.07
Access circuit primary patency (PP) over 12 mos, % (95% CI)	36.4 (24.8–48.1)	25.2 (16.0–35.5)	0.044
Number of repeat interventions over 12 mos; median (IQR)	1 (0–1)	1 (0–2)	0.047
Number of repeat interventions; *n* (%)			
0	38 (45.78)	29 (33.33)	
1	26 (31.33)	26 (29.89)	
2	13 (15.66)	23 (26.44)	
3	4 (4.82)	7 (8.05)	
4	1 (1.20)	2 (2.30)	
5	1 (1.20)	0 (0.00)	
Brachial artery flow, ml/min			
3-mo; median (IQR)	733.5 (*n* = 76) (536.0–978.7)	682.8 (*n* = 70) (496.7–956.7)	0.39
6-mo; median (IQR)	610.0 (*n* = 52) (386.5–857.8)	631.3 (*n* = 45) (496.7–826.0)	0.48
12-mo; median (IQR)	643.0 (*n* = 29) (506.7–973.0)	710.2 (*n* = 22) (618.3–948.7)	0.35
Outflow Vein flow, ml/min			
3-mo; median (IQR)	576.5 (*n* = 76) (381.0–877.5)	590.2 (*n* = 70) (356.3–795.3)	0.72
6-mo; median (IQR)	485.7 (*n* = 52) (305.5–777.8)	563.3 (*n* = 45) (394.3–818.0)	0.20
12-mo; median (IQR)	619.0 (*n* = 29) (373.3–731.0)	614.7 (*n* = 22) (521.3–836.3)	0.51

CI, confidence interval; IQR, interquartile range; ITT, intention-to-treat; PP, per-protocol.

**Figure 3:**
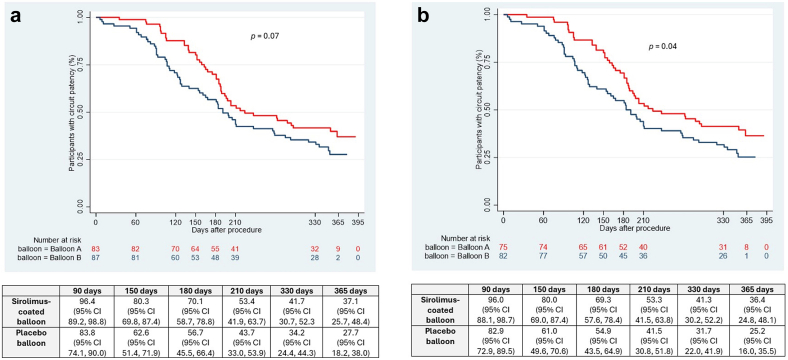
(a) Kaplan-Meier analysis of access circuit primary patency during 12 months after index intervention (ITT analyses). (b) Kaplan-Meier analyses of access circuit primary patency during 12 months after index intervention (PP analyses). CI, confidence interval; ITT, intention-to-treat; PP, per-protocol.

In Figure 3a, we show that using the ITT approach, there was no significant difference in the ACPP between SCB and placebo balloons over the 12 months (*P* = 0.07). The beneficial effect of SCB did not persist up to 12 months of follow-up on ITT analysis. At 12 months, 37.1% (95% CI: 25.7–48.4) of the patients treated with SCB remained patent whereas 27.7% (95% CI: 18.2–38.0) of the patients treated with placebo balloons were patent using the ITT approach. However, on the PP approach, there was a significant difference in the ACPP between SCB and placebo balloon over the 12 months (*P* = 0.04). At 365 days, 36.4% (95% CI: 24.8–48.1) of the patients treated with SCB remained patent whereas 25.2% (95% CI: 16.0–35.5) of the patients treated with placebo were patent (Figure 3b).

**Figure 4:**
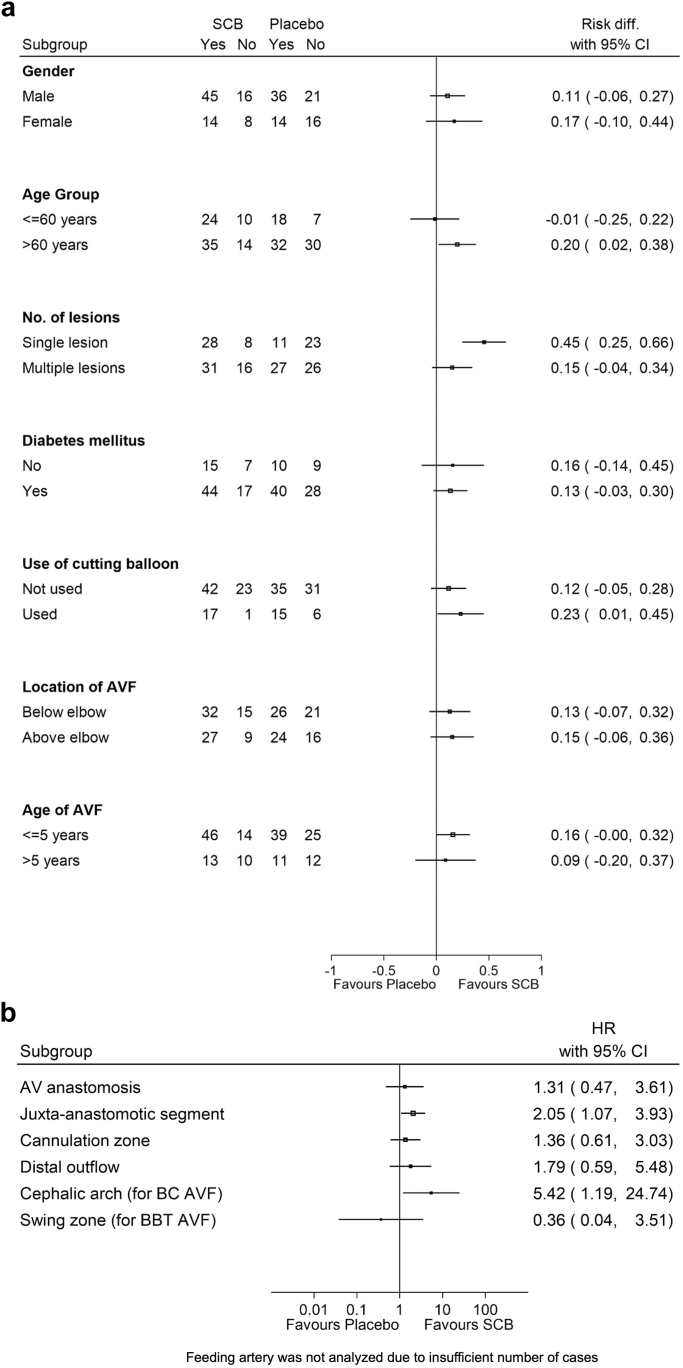
(a) Subgroup analysis of primary end points. (b) Subgroup analysis of lesion specific patency. AV, arteriovenous; AVF, arteriovenous fistula; BBT, brachiobasilic transposition; BC, brachiocephalic; CI, confidence interval; SCB, sirolimus-coated angioplasty balloon.

Subgroup analyses were conducted in gender, age, diabetes, age of AVF, location of AVF, and use of cutting balloon (Figure 4a). Forest plots of risk differences demonstrated a consistent reduction in the risk of loss of ACPP over 6 months with SCB, with point estimates favoring SCB across selected baseline characteristics of the study cohort. In exploratory subgroup analysis by lesion location (Figure 4b), hazard ratios for loss of ACPP over 6 months on ITT analysis consistently favored SCB across all evaluable lesion types. Confidence intervals were wide and crossed unity, reflecting limited power for subgroup comparison.

### Safety End Points

In Table 3, we show that access circuit–related adverse events within 30 days occurred in 2.4% of patients in the SCB group and 0% in the placebo group, with a risk difference of 2.4% (95% CI: −1.9 to 8.4) based on the ITT analysis. Similarly, the risk difference was 2.6% (95% CI: −0.02 to 0.1), based on PP analyses.

**Table 3:** Safety end points analyses

End point	Sirolimus-coated balloon	Placebo balloon	Difference^a^ (95% CI)	*P-*value^b^
ITT analyses				
Primary safety end point				
Adverse events related to treatment within 30 d – no./total no. (%)	2/83 (2.4)	0/86 (0.0)	2.4 (−1.9 to 8.4)	0.039
Bleeding/hematoma	1/83 (1.2)	0/86 (0.0)		
Venous rupture	1/83 (1.2)	0/86 (0.0)		
Secondary safety end points				
Thrombolysis within 30 d	0/83 (0.0)	4/86 (4.7)	−4.7 (−9.2 to −0.2)	0.023
Death within 30 d	0	0	-	-
PP analyses				
Primary safety end point				
Adverse events related to treatment within 30 d – no./total no. (%)	2/78 (2.6)	0/84 (0.0)	2.6 (−0.02 to 0.1)	0.049
Bleeding/hematoma	1/78 (1.3)	0/84 (0.0)		
Venous rupture	1/78 (1.3)	0/84 (0.0)		
Secondary safety end points				
Thrombolysis within 30 d	0/78 (0.0)	4/84 (4.8)	−4.8 (−9.3 to −0.2)	0.026
Death within 30 d	0	0	-	-

CI, confidence interval; ITT, intention-to-treat; no., number.

Excludes those who died or withdrew within 30 d.

a Difference refers to sirolimus-coated balloon – placebo balloon.

b *P*-value for noninferiority for the primary safety end point (adverse events) was based on the Farrington–Manning noninferiority test with a margin of 7.5%. *P*-value for the secondary safety end point was based on 1-sided test of 2 proportions. Significance level was set at 5% for 2-sided test and 2.5% for 1-sided test.

In the ITT analysis, no patients in the SCB group required thrombolysis for thrombosed access within 30 days of intervention, compared with 4 of 86 surviving patients in the placebo group (Table 3). The proportion of patients who required thrombolysis within 30 days in the SCB group was significantly lower than in the placebo group (*P* = 0.02). No death were reported within 30 days in either the SCB or placebo balloon treated groups.

## Discussion

Nearly 4 million people in the world are living on kidney replacement therapy, and hemodialysis accounts for 89% of all dialysis patients in the world.^40^ Due to the scarcity of organ transplants, many patients remain on hemodialysis for many years. In Singapore, the average waiting time for a deceased donor kidney transplant is 9 years and hemodialysis is the predominant form of kidney replacement therapy.^41^ Maintaining functioning access is therefore critical to ensure the continual delivery of life-saving treatment for patients on hemodialysis. Access dysfunction is the leading cause of hospitalization and morbidities in patients on hemodialysis, and part of the problem is the poor patency associated with the current standard of care.^42^ Recurrence is high with 1-year post angioplasty patency rates of 40% to 60% in the patients with dysfunctional AVF who were treated with PBA.^43^

This study, to evaluate the effectiveness and safety of SCB for treating stenoses in dysfunctional AVFs was conducted in Singapore, which has a multiethnic population. The procedures were performed by interventional radiologists, nephrologists, and vascular surgeons across 3 teaching hospitals with outcomes adjudicated by the respective site principal investigators. The SCB outperformed the placebo balloon in terms of the percentage of participants whose AVFs remained free from clinically indicated reintervention at 6 months postprocedure. In addition, adverse events related to treatment within 30 days were infrequent in both groups, and no deaths occurred within 30 days in either arm. The findings from this trial are promising and provide evidence of the short-term benefits and safety of this device. Due to deaths and withdrawals from the study, the beneficial effects of SCB on the ACPP for the cohort were diminished at 12 months. In patients who completed 12-month follow-up, those who received SCB had significantly better ACPP than those who received placebo. The statistical significance of the difference in patency on PP analysis was 0.04, indicating that the statistical significance might be fragile and hence sensitive to outliers.

Direct comparison of our results with randomized controlled trials of PCB in dialysis access, though relevant, should be made cautiously. Major PCB trials used target lesion primary patency as the primary efficacy end point, whereas our study used ACPP as the primary end point. In addition, the PCB studies focused on single or tandem lesions that could be treated with a single PCB, whereas our study included single or multiple stenoses without restriction on the number of SCBs used. Across these PCB trials, 6-month target lesion patency in the PCB arms ranged from 71% to 91%; whereas reported 6-month ACPP rates, where available, ranged from 62% to 82%.^5^^,^^19^, ^20^, ^21^, ^22^^,^^44^^,^^45^ Although the 6-month ACPP rates of 70.1% in the SCB group and 56.7% in the placebo group in our study fell within the range reported in previous PCB studies, this should not be interpreted as demonstrating comparable efficacy because of important differences in end-point selection and lesion complexity. These differences limit direct cross-trial comparison and may partly explain the lower 12-month ACPP observed in our study. The lower 12-month ACPP observed with SCB may not imply inferior intrinsic potency of sirolimus relative to paclitaxel. In the coronary literature, sirolimus-eluting stents have generally outperformed paclitaxel-eluting stents,^46^ and second generation coronary stents that are limus-based have been shown to be superior to paclitaxel eluting stents.^47^^,^^48^ Rather, pharmacologic differences may be relevant in the balloon-delivery context: sirolimus has lower lipophilicity and tissue retention than paclitaxel, so a single SCB application may achieve sufficient tissue levels to suppress neointimal hyperplasia at 6 months but not maintain inhibitory concentrations through 12 months. This raises the hypothesis that repeat SCB dosing could extend durability, a strategy that warrants prospective evaluation.

Although a 2019 meta-analysis did not demonstrate an increase in short- or midterm mortality associated with PCB in dialysis access interventions,^49^ historical concerns regarding potential late mortality with paclitaxel-coated devices remain part of the broader peripheral arterial disease literature. More recently, in a large registry-based randomized trial of infrainguinal endovascular treatment for intermittent claudication, higher 5-year mortality was observed with drug-coated devices, although overall, all-cause mortality over 7.1 years did not differ significantly.^50^ With the ongoing concerns and debate over paclitaxel-coated devices, SCB may represent an alternative drug-coated balloon platform, especially in patients with multiple stenoses that require the use of >1 drug-coated balloon. However, the present study was not designed to determine whether SCB offers any long-term safety advantage over PCB. Our study nevertheless demonstrated that, in addition to good-quality vessel preparation with standard balloon angioplasty and meticulous attention to residual stenosis, the use of SCB as an adjunctive therapy for dysfunctional AVFs following successful PTA can prolong the time to the next intervention. Larger, longer-term efficacy and safety data are needed to confirm these observations. In addition, the economic implications of SCB use, including device cost, reintervention burden, and overall healthcare utilization, should be evaluated in future studies.

This trial has several limitations. Because the SCB differs in appearance from a standard balloon, a true double-blinded trial design to blind the interventionalists and trial coordinators was not possible. Although ACPP is a clinically relevant end point, it is vulnerable to clinician-dependent variability. Despite the use of standardized reintervention criteria and having a central review by the clinical evaluation committee, some degree of ascertainment bias cannot be excluded. The study was conducted in Singapore and the generalizability of our findings to other countries’ populations may be hampered by potential differences in dialysis vintage, vessel sizes, and characteristics of stenoses. The use of cutting balloons as part of the protocol to achieve adequacy of vessel preparation before the application of SCB or placebo balloons was part of our standard-of-care treatment algorithm based on our previous randomized controlled trial.^51^ In this study, the number of patients who received cutting balloons was similar in both groups and the application of SCB had an additional beneficial effect over and above the effects of cutting balloons on the ACPP. The safety and effectiveness of SCB for treating central vein obstruction and in-stent restenosis were not evaluated because none of the patients in the trial had significant central vein stenosis and patients with previous stents within the dialysis circuit were excluded from this trial.

Nevertheless, within the limitations of our study, treatment of dysfunctional AVFs with SCB provided superior 6-month ACPP compared with standard balloon angioplasty alone. Short-term safety findings were reassuring, although long-term safety cannot be determined from this study. A larger international study is being planned to confirm our findings.

## Appendix

### List of the IMPRESSION investigators

Darius Kang Lie Aw (MD, MMed, FRCSEd), Shaun Xavier Ju Min Chan (MBBS, PGDipECHO, FRCR), Siew Ping Chng (MB ChB, MRCS, FAMS), Edward Tieng Chek Choke (MBBS, PhD, FRCS), Tze Tec Chong (MBBS, FACS, RPVI), Jasmine Ming Er Chua (MBBS, MMed, FRCR), Karthikeyan Damodharan (MBBS, MRCP, FRCR, EBIR, FCIRSE, FAMS), Rajesh Babu Dharmaraj (MBBS, MMed, MRCS, FRCS), Chye Chung Gan (MBBS, MRCP, FRCP, ESENeph, AM), Apoorva Gogna (MBBS, FRCR, EBIR, FCIRSE, FAMS), Anil Gopinathan (MBBS, FRCR, FAMS), Farah G. Irani (MBBS, MD, FRCR, FAMS), Pradesh Kumar Kutty Krishnan (MB ChB, MRCS, FRCR), Kristen Alexa Lee (MD, board-certified in Diagnostic Radiology and Interventional Radiology), Sum Leong (MB BCh BAO, MSc, MRCS, FFR(RCSI)), Richard Hoau Gong Lo (MBBS, FRCR, FAMS), Stanley Eu Kwang Loh (MBChB, MMed, MRCS, FRCR), Jun Jie Ng (MBBS, MMed, MRCS, FRCSEd), Shao Jin Ong (BSc (Hons), MBBS, PhD, FRCR, FAMS), Suh Chien Pang (MBBS, MRCP), Ankur Patel (BMedSc, MB ChB, MMed, MRCS, FRCR, FAMS), Jackie Ho Pei (MBBS, FRCS (Edin), FCS (HK), FHKAM (Gen Surg)), Chandramohan Sivanathan (MBBS, MRCS, FRCR, EBIR), Alfred Bingchao Tan (BSc MedSci (Hons), MB ChB, MRCS, FRCR, EBIR), Alexander Sheng Ming Tan (MBBS, MMed, MRCS, FRCR), Bien Soo Tan (MBBS, FRCR, FAMS), Chee Wooi Tan (MBBS, MRCP), Chieh Suai Tan (MBBS, MRCP, FRCP, FAMS), Ru Yu Tan (MBBS, MMedStats, MRCP, FAMS), Zehao Tan (MBBS, MMed, FRCR), Tjun Yip Tang (MA, MB BChir, MD, FRCS, FAMS), Sonam Tashi (MBBS, MMed, FRCR, FAMS), Hsien Ts’ung Tay (MBBS, MSc, MRCS, FRCS), Jia Sheng Tay (MBBS, MMed, FRCSEd), Kiang Hiong Tay (MBBS, FRCR, FSIR, FAMS), Alvin Ren Kwang Tng (MBBS, MRCP), Luke Han Wei Toh (MBBS, FRCR, FAMS), Chow Wei Too (MBBS, MMed, FRCR, FAMS), Nanda Kumar Karaddi Venkatanarasimha (MBBS, MRCP, FRCR, FRANZCR, EBIR, FCIRSE), Kalpana Vijaykumar (MBBS, MMed, FRCSEd), Mark Qi Wei Wang (MBBS, FRCR, FAMS), Julian Chi Leung Wong (MB ChB, FRCS, PGDMedEd, FAMS), Chen Xinquan (BSc (Hons)), Hao Yun Yap (MBBS, MMed, MRCSEd, FRCSEd), Gary Sem Wye Yoong (MBBS, MMed, FRCR), Kun Da Zhuang (MBBS, MMed, FRCR).

## Disclosure

All the authors declared no competing interests.

## Supplementary Material

Supplementary File (PDF) **Table S1.** Indications and types of repeat intervention that resulted in loss of access circuit primary patency. CONSORT Checklist.

## Footnotes

Supplementary File (PDF)
**Table S1.** Indications and types of repeat intervention that resulted in loss of access circuit primary patency. CONSORT Checklist.
